# Subcutaneous adipose tissue expansion mechanisms are similar in early and late onset overweight/obesity

**DOI:** 10.1038/s41366-022-01102-6

**Published:** 2022-02-28

**Authors:** Peter Arner, Daniel P. Andersson, Erik Arner, Mikael Rydén, Alastair G. Kerr

**Affiliations:** 1 Department of Medicine (H7), Karolinska Institutet at Karolinska University Hospital Huddinge, Center for Metabolism and Endocrinology, 14186 Stockholm, Sweden; 2 GSK, Gunnels Wood Rd, Stevenage, SG1 2NY United Kingdom

**Keywords:** Obesity, Obesity

## Abstract

**Background/objective:**

The development of overweight/obesity associates with alterations in white adipose tissue (WAT) cellularity (fat cell size/number) and lipid metabolism, in particular lipolysis. If these changes differ between early/juvenile (EOO < 18 years of age) or late onset overweight/obesity (LOO) is unknown and was presently examined.

**Subjects/methods:**

We included 439 subjects with validated information on body mass index (BMI) at 18 years of age. Using this information and current BMI, subjects were divided into never overweight/obese (BMI < 25 kg/m^2^), EOO and LOO. Adipocyte size, number, morphology (size in relation to body fat) and lipolysis were determined in subcutaneous abdominal WAT. Body composition and WAT distribution was assessed by dual-X-ray absorptiometry.

**Results:**

Compared with never overweight/obese, EOO and LOO displayed larger WAT amounts in all examined depots, which in subcutaneous WAT was explained by a combination of increased size and number of fat cells in EOO and LOO. EOO had 40% larger subcutaneous fat mass than LOO (*p* < 0.0001). Visceral WAT mass, WAT morphology and lipolysis did not differ between EOO and LOO except for minor differences in men between the two obesity groups. On average, the increase in BMI per year was 57% higher in subjects with EOO compared to LOO (*p* < 0.0001).

**Conclusion:**

Early onset overweight/obesity causes a more rapid and pronounced accumulation of subcutaneous WAT than adult onset. However, fat mass expansion measures including WAT cellularity, morphology and fat cell lipolysis do not differ in an important way suggesting that similar mechanisms of WAT growth operate in EOO and LOO.

## Introduction

White adipose tissue (WAT) grows by increasing the size and/or number of fat cells [1]. Earlier studies suggested that WAT expands during childhood/adolescence due to a combination of elevated fat cell size and number, whereas in adulthood the increase is solely due to enlargement of pre-existing fat cells as reviewed [2, 3]. This idea has recently been challenged by studies demonstrating that body weight gain or regain over time in adults also involves increases in fat cell number [4–6]. The dynamic nature of WAT cellularity has clinical consequences as it may cause the development of two distinct morphology phenotypes, namely WAT hypertrophy with fewer but larger cells or WAT hyperplasia with many small cells [2, 3]. The former has been demonstrated to be the more pernicious type of expansion [1–3].

Another aspect of WAT expansion is the ability to mobilize lipids through hydrolysis (lipolysis) of triglycerides in fat cells. Numerous cross-sectional studies have demonstrated that catecholamine stimulated lipolysis is decreased in subjects with excess body fat as reviewed [7, 8]. In one recent prospective study such a defect was found to be an independent risk factor for future body weight gain [9]. Finally, weight cycling with periods of weight loss and regain may influence the development of overweight/obesity in adulthood as discussed [10, 11].

Very little is known about the WAT phenotype in subjects who develop excess body fat early or late in life. In patients with obesity scheduled for bariatric surgery, a higher body mass index (BMI) was observed if they had juvenile as compared to adult-onset obesity [12, 13]. We hypothesized that the mechanisms behind adipose expansion (WAT cellularity, morphology, lipolysis, or body weight fluctuations) are similar between these two forms of overweight/obesity in spite of a higher body fat mass in early onset overweight/obesity. To answer these important questions, we investigated 439 adult subjects where information about their body weight at 18 years of age and their highest/lowest body weight since that time was available. Fat cell size and number (cellularity), morphology (hypertrophy/hyperplasia) and spontaneous (basal) or catecholamine-stimulated lipolysis were determined in abdominal subcutaneous WAT. In addition, body fat distribution was measured.

## Research design and methods

### Subjects

The subjects were adults examined during 1996 to 2020 and included here for studies of the impact of overweight/obesity onset on WAT. They were asked about body weight at 18 years of age. There is no consensus on how to define adult/late onset of excess body fat. We used 18 years old as a threshold. It marks the end of adolescence in most Western societies and there is usually no important increase in BMI after this age. We included those who made notes on this body weight and also about highest/lowest body weight from 18 years of age and onwards. The current height was used to calculate BMI at 18 years of age and the highest/lowest values from 18 years of age. Based on BMI at 18 years of age and at the laboratory examination they could be classified as being (a) lean on both occasions (BMI < 25 kg/m^2^, *n* = 88), (b) overweight or obese on both occasions, termed early onset (BMI ≥ 25 kg/m^2^, *n* = 96) or EOO, (c) changing from lean at 18 years to overweight/obese at current examination, termed late onset (*n* = 255) or LOO (d) or changing from overweight/obese to lean (*n* = 5). Because the focus was on current overweight/obesity and the last group was considered too small for analysis it was excluded from the study. Other exclusion criteria were severe chronic disorder and type 1 diabetes. Type 2 diabetes was present in 91 subjects and treated with diet, oral antidiabetic agents excluding thiazolidinediones or insulin (in four patients). Twenty subjects with hypertension were on beta-blockers. The reliability of self-reported BMI was validated as follows. During 2012 until 2020 we asked 259 adults who were scheduled for a research examination at our laboratory about current body weight using measures at home. This weight was compared with the examined body weight at the laboratory and used for assessment of the accuracy of home weighing. Additionally, twenty-nine subjects returned to the laboratory for studies not related to the current one with a 2–20-year interval (median 8 years). They were again asked for body weight at 18 years of age to validate the accuracy of this information. The study was approved by the local committee on ethics. It was explained in detail to each subject and written informed consent was obtained.

### Clinical examination

The subjects came to the laboratory in the morning after an overnight fast and a venous blood sample was obtained for routine clinical chemistry measures. In 41 subjects we also measured plasma leptin with a commercial kit (Linco Research Inc. ST Charles, MO, USA). Height, body weight and circumferences of waist and hip were determined. This was followed by measures of body composition with dual X-ray absorptiometry (DEXA) using Lunar I DXA, Encore Version 16 SP1 (GE Health Care, Stockholm, Sweden), which is approved by the Food and Drug Administration in USA to measure regional- and total body fat. The android region was defined inferiorly at the pelvis cut line, superiorly by 20% of the distance between neck cut and the pelvis and laterally at the arm cut lines. The gynoid region was defined superiorly below the pelvis cut line by 1.5 times the height of the android region, inferiorly below the superior line by two times the height of the android region and laterally at the outer leg cut lines. Using the CoreScan software (GE Health Care, Stockholm, Sweden) to analyze DXA measurements, the amount of visceral adipose tissue (EVAT) was estimated in the android region. The CoreScan software uses measurement of total body fat in the android region and estimated subcutaneous fat in the android region (ESAT) to estimate EVAT with the following formula: EVAT = total body fat in the android region – ESAT. EVAT estimation has a very high concordance (*r*^2^ = 0.96) with measurement of visceral adipose tissue using computed tomography, which is the gold standard method, as described previously [14]. Since both android fat mass and EVAT are valid measures, ESAT could be calculated as total android fat minus EVAT. Additionally, a subcutaneous abdominal fat biopsy was obtained by needle aspiration from the ESAT area. In 215 subjects, a 2 h hyperinsulinemic euglycemic clamp was performed exactly as described [15]. The whole-body uptake of glucose (M) during the last hour of the clamp was determined and related to lean body mass.

### Adipose tissue examinations

Isolated fat cells were prepared and incubated in vitro as described [16] for 2 h in the absence (basal) or presence of either the natural catecholamine, noradrenaline, or the synthetic beta-adrenoceptor selective catecholamine termed isoprenaline. Different concentrations of the catecholamines were used in duplicate (10^−12^ to 10^−4^ mol/l) and results with maximum effective concentration were used in the analyses. At the end of incubation the medium was removed for determinations of glycerol (lipolysis index) and lipids were extracted from the cells in the incubated sample. The release of glycerol in these conditions is linear with time for at least 4 h. We have discussed in detail the validity of using the present fat cell protocol and glycerol as a lipolysis indicator [17]. There is no consensus on how to express lipolysis rates. Therefore, we used the two most common denominators which are per lipid weight or number of fat cells in the incubated samples. The former expression is not influenced by fat cell size as opposed to the second one. On one aliquot of the isolated fat cells the diameter of 100 randomly selected fat cells was measured. These data were used to determine average lipid weight and size of the fat cells using well established formulas. Details of the method and discussion of its validity have been presented before [18]. The same three technicians performed the fat cell studies throughout the study. The number of fat cells incubated was determined by dividing the total lipid weight of the sample by the mean fat cell weight. The total number of fat cells in ESAT was obtained by dividing ESAT weight with mean fat cell weight. Adipose tissue expands by increasing the size (hypertrophy) and/or number (hyperplasia) of the fat cells. By determining the curve-linear relationship between fat mass and fat cell size the morphology (hypertrophy versus hyperplasia) can be determined as discussed in detail [18, 19]. For ESAT the relationship fits the formula V = (a × m)/(1 + b × m) were V is mean fat cell volume, m is amount of ESAT and a and b are variables obtained by fitting the formula to subject data [18]. The difference between measured fat cell volume and the expected fat cell volume from the mean curve fit is termed delta and indicates the morphology of ESAT as discussed in detail [18]. A positive value (above the line) indicates hypertrophy and a negative (below the line) indicates hyperplasia.

### Statistical methods

Values are mean ± SD in text and tables and box plots or individuals in figures. Values for lipolysis were normalized by (10)log transformation. The primary comparison was between all three BMI groups. When this was statistically significant a secondary comparison between the two overweight/obese groups was made. Continuous variables were compared by analysis of variance (ANOVA) as first and unpaired t-test as second comparison. Because we only compared EOO and LOO in second comparison there was no adjustment for multiple comparison. Category variables were compared by Chi-Square. The relative roles of multiple explanatory factors to predict the outcome of a response variable was tested using multiple regression. For clinical variables, these regression models included age, sex, body mass index body mass index at 18 years of age and onset of overweight/obesity. For lipolysis, the regression model included onset of obesity/overweight percentage body fat, waist-to-hip ratio, age, sex and fat cell volume as independent factors. The % variation between two measures of body weight was calculated as net difference divided by mean values times 100. All tests were two-sided and *p* < 0.05 was considered as statistically significant.

## Results

The clinical data are recorded in Table 1. Both overweight/obesity conditions displayed the same type of differences in fasting glucose and triglycerides when compared with always lean subjects. However, EOO were younger and had higher BMI than LOO. The temporal changes in BMI in the two overweight/obesity groups were further analyzed. Delta BMI (current minus at 18 years of age) was 8.7 ± 6.2 kg/m^2^ in EOO and 10.5 ± 6.0 kg/m^2^ in LOO (*p* = 0.004). When delta BMI was divided by observation time (current age – 18) it was 0.58 ± 0.63 kg/m^2^ /year in early onset and 0.37 ± 0.36 kg/m^2^/year in late onset (*p* < 0.0001). Thus, the BMI difference between groups was most likely due to a higher starting value plus greater BMI increase per year during adulthood in those with early onset overweight/obesity. The two overweight/obesity groups differed also somewhat with respect to fasting insulin and HDL-cholesterol. The nature of the differences was further analyzed by multiple regression using a model including sex, age, and BMI, as these factors differed significantly between the two overweight/obesity groups. In the regression model, onset of overweight/obesity did not contribute significantly to the variations between subjects in insulin or HDL-cholesterol (Table S1). As expected, both forms of overweight/obesity displayed decreased in vivo insulin sensitivity, but the level of insulin resistance was not different between EOO and LOO. The occurrence of type 2 diabetes was slightly more frequent in late *vs*. early onset of overweight/obesity (*p* = 0.034). On a few subjects we also measured fasting leptin. Values (ng/ml) were 9 ± 3, 47 ± 17 and 65 ± 36 in never overweight/obese (*n* = 6), LOO (*n* = 20) and EOO (*n* = 15), respectively (ANOVA: p-value 0.0002; p-value for LOO *vs* EOO = 0.04).

**Table 1:** Clinical characteristics.

Phenotype	Never overweight or obesity (A)	Adult overweight or obesity (B)	Early overweight or obesity (C)	ABC ANOVA or Chi-Square, *p*-value	C-B, t-test or Chi-Square, *p*-value
Males/females	36/52	103/152	27/71	0.065	–
Age (years)	53 ± 12	54 ± 11	42 ± 13	<0.0001	<0.0001
Current BMI (kg/m^2^)	23.0 ± 1.6	32.2 ± 6.0	38.2 ± 6.4	<0.0001	<0.0001
BMI at age 18 years (kg/m^2^)	21 ± 2	22 ± 2	30 ± 4	<0.0001	–
Diabetes (yes/no)	14/24	63/192	14/84	0.045	0.034
Hypertension (yes/no)	20/68	97/158	29/69	0.022	0.14
Dyslipidemia (yes/no)	7/81	30/225	8/90	0.45	–
S-insulin (log mU/l)	5.4 ± 2.6	11.1 ± 14.1	14.1 ± 9.1	<0.0001	0.001
P-glucose (mmol/l)	5.5 ± 1.0	6.2 ± 1.8	5.6 ± 1.6	0.0009	0.06
P-total cholesterol (mmol/l)	4.7 ± 1.0	4.8 ± 1.1	4.6 ± 0.9	0.10	–
P-HDL-cholesterol (mmol/l)	1.59 ± 0.45	1.30 ± 0.33	1.18 ± 0.28	<0.0001	0.007
P-triglycerides (mmol/l)	0.9 ± 0.5	1.4 ± 0.8	1.5 ± 0.8	<0.0001	0.54
M (mg/kg lean body mass/min)	13.1 ± 2.5	10.2 ± 3.5	9.8 ± 3.7	<0.0001	0.49

Values are mean ± SD. The initial comparison was for overall difference. If significant, secondary comparison was between the two overweight/obesity groups. HDL High density lipoprotein. HOMA-IR Homeostasis model assessment of insulin resistance. M Whole body glucose uptake during second hour of a hyperinsulinemic euglycemic clamp, which was performed in 35, 110 and 66 subjects in A, B and C, respectively. P Fasting plasma. S Fasting serum.

**Fig. 1:**
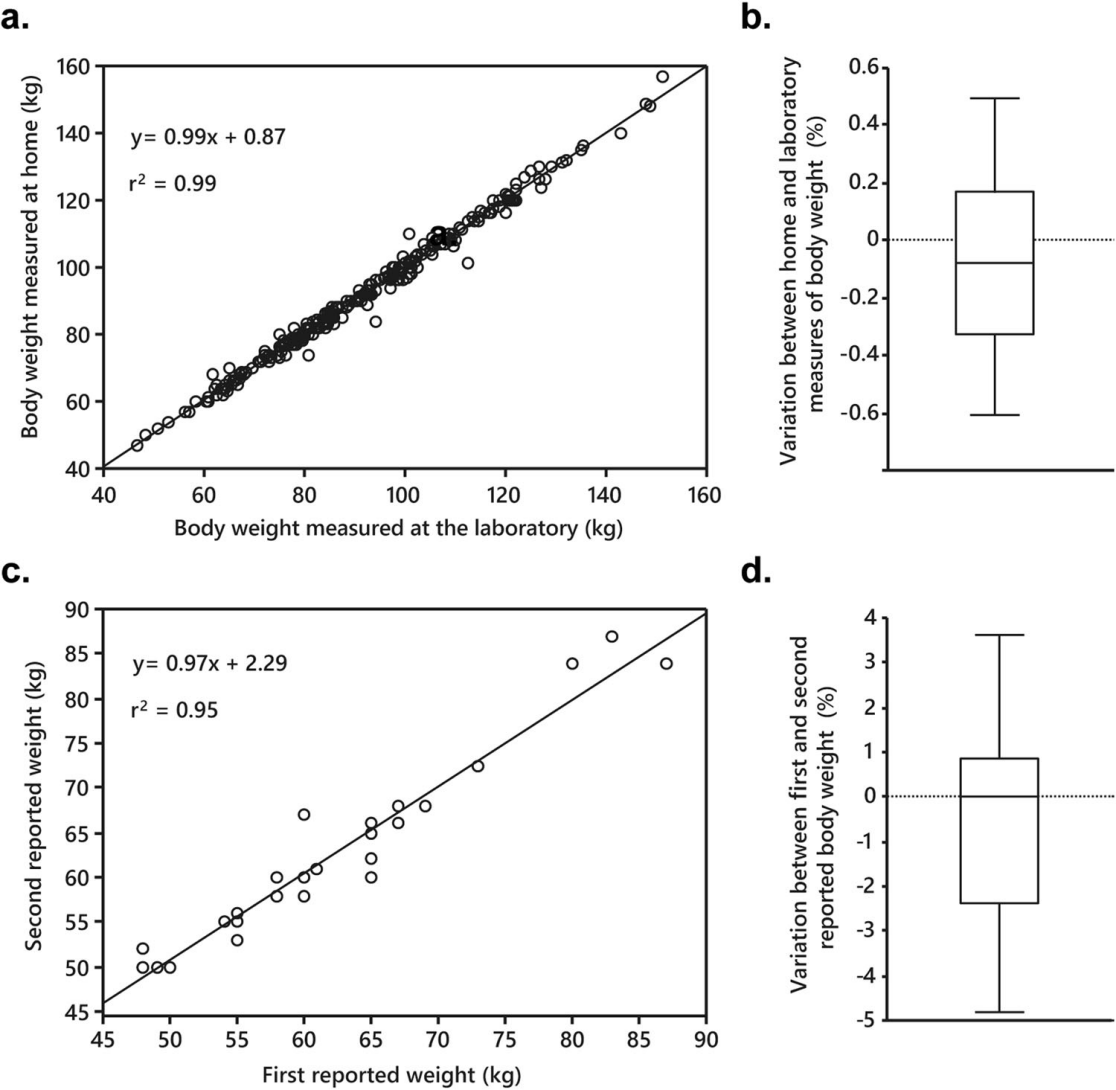
Validation of self-reported body mass index (BMI). **a** Is the relationship between current BMI measured at home and at the laboratory. **b** Is the variance between home and laboratory measures. **c** Is the comparison between BMI information at 18 years of age obtained at two occasions. **d** Is variation between the two reports. Linear regression is shown in (**a**) and (**c**) indicating the square of regression coefficients, intercepts and slopes.

We validated self-reported BMI. The current determination of body weight at the laboratory and at home was highly correlated (Fig. 1a, b, *r*^2^ = 0.99). There was also very little deviation in the reported BMI at 18 years of age when determined at two different occasions with a median interval of 8 years (Fig. 1c, d). The variation in body weight between the two measures in both validation studies was <1% (Fig. 1b, d). The slopes were near one and intercepts not significantly different from zero in either of the regression analyses.

Data with body composition are recorded in Table 2. As expected, total body fat mass was increased in both overweight/obesity groups but almost 40% more so in EOO. Waist, hip, and android or gynoid fat depots were larger in EOO compared to LOO, but the two groups did not differ in visceral fat mass or in the waist-to-hip ratio. Also as expected, lean body mass was increased in overweight/obesity and slightly more so in EOO (5%). Taken together these data suggest that higher BMI in EOO than LOO is above all due to larger amounts of lean body mass and subcutaneous WAT.

**Table 2:** Body composition.

Phenotype	Never overweight/obese A	Adult Overweight/obese B	Early Overweight/obese C	*p*-value A,B,C	*p*-value B-C
Hip circumference (cm)	97 ± 6	112 ± 14	122 ± 13	<0.0001	<0.0001
Waist circumference (cm)	85 ± 7	109 ± 14	120 ± 14	<0.0001	<0.0001
Waist-to-hip (ratio)	0.88 ± 0.07	0.97 ± 0.07	0.98 ± 0.08	<0.0001	0.31
Body weight (kg)	69 ± 10	94 ± 17	109 ± 19	<0.0001	<0.0011
Lean body mass (kg)	47.0 ± 9.7	52.1 ± 8.4	54.9 ± 9.5	<0.0001	0.014
Total fat mass (kg)	19.5 ± 4.8	36.7 ± 13.8	50.7 ± 15.0	<0.0001	<0.0001
Per cent body fat (%)	29.7 ± 7.3	41.9 ± 9.3	47.4 ± 8.2	<0.0001	<0.0001
Android fat (kg)	1.6 ± 0.7	4.0 ± 1.6	5.2 ± 1.6	<0.0001	<0.0001
Gynoid fat (kg)	3.3 ± 1.1	6.0 ± 2.5	8.1 ± 3.1	<0.0001	<0.0001
Visceral fat (kg)	0.7 ± 0.5	1.9 ± 1.0	2.1 ± 1.0	<0.0001	0.26

Values are mean ± SD. They were first compared by analysis of variance. If this was statistically significant a secondary comparison was made using unpaired *t*-test.

**Fig. 2:**
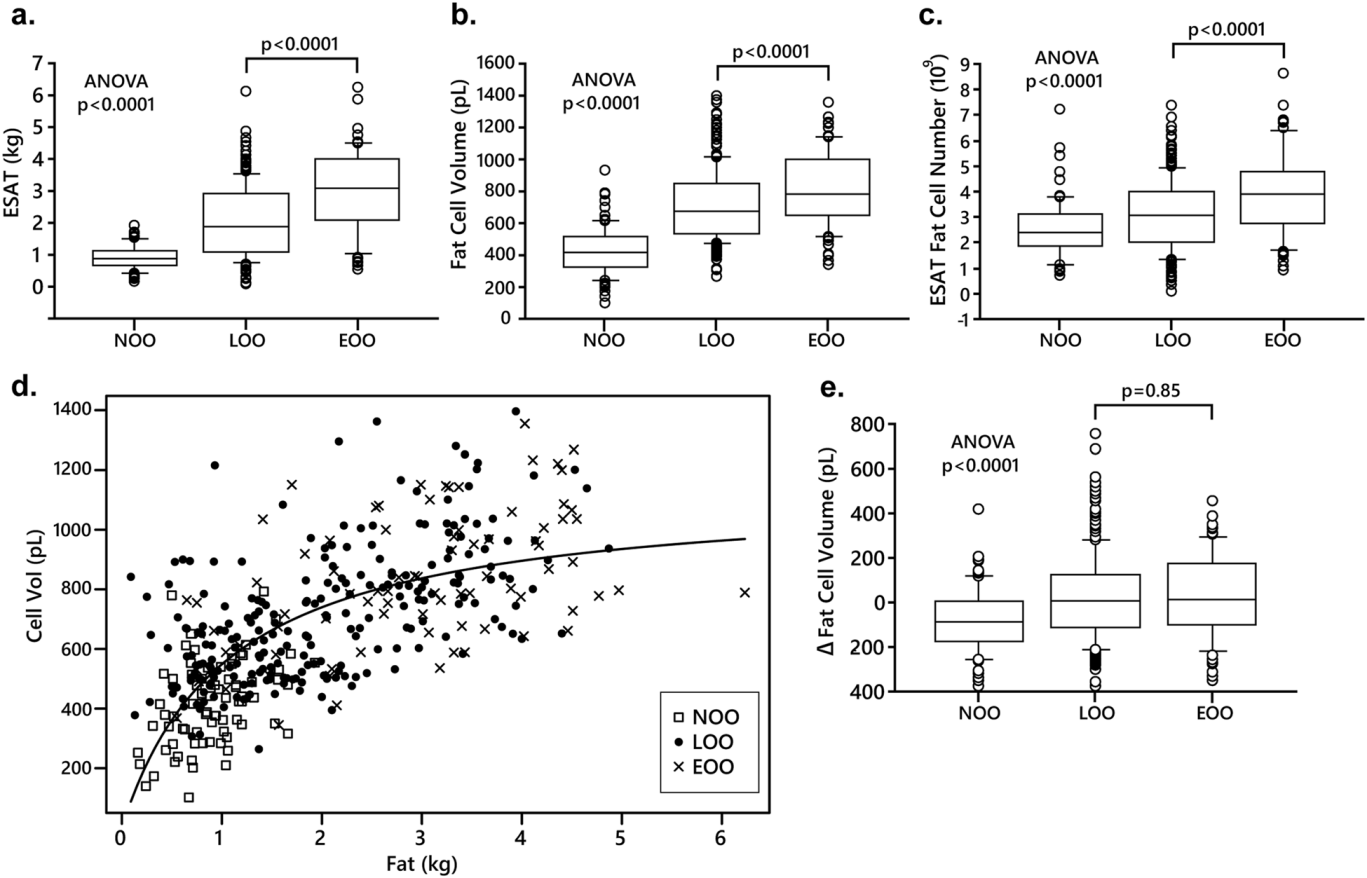
Findings with abdominal subcutaneous adipose tissue (ESAT). **a** Is ESAT mass. **b** Is mean fat cell volume. **c** Is number of fat cells in ESAT. **d** Is the curve-linear relationship between fat cell volume and ESAT mass. **e** Is ESAT morphology (delta value) calculated from the results in (**d**). Values are individual in (**d**) and box plots otherwise with outliers plotted as individual points. The three phenotypes were compared overall by ANOVA. The two overweight/obesity groups were compared by unpaired t-test. NOO never overweight/obesity, EOO Early onset overweight/obesity, LOO Late onset overweight/obesity.

The findings with ESAT are shown in Fig. 2. As expected from the results in Table 2 this fat mass was increased in both forms of overweight/obesity and significantly more so in the early onset form (Fig. 2a). The increased ESAT mass could be explained by a combination of having larger and more fat cells than in the always lean condition (Fig. 2b, c). The increases in both fat cell measures were significantly more pronounced in the early onset form of overweight/obesity. However, in all subjects put together there was a strong positive relationship between the rate of BMI increase over time (current BMI minus BMI at 18 years divided by observation time) and fat cell number or fat cell volume (*r*^2^ = 0.21–0.424 *p* < 0.0001). These relationships were also observed when never overweight/obese, EOO and LOO were investigated separately (*r*^2^ ≥ 0.10; *p* ≤ 0.02). The curve linear relationship between ESAT mass and fat cell volume is depicted in Fig. 2d. Visually, lean subjects tended to be more frequently positioned below the fitted line. The morphology of ESAT was quantified in Fig. 2e showing the delta fat cell volume values. Small but statistically significant differences between always lean and LOO/EOO were observed. The always lean phenotype showed modest hyperplasia (negative mean value) and the two overweight/obesity phenotypes had slight hypertrophy, i.e., positive mean values, but there were no differences in morphology (i.e., delta) between the latter two phenotypes. Taken together the data suggest that the increased growth of WAT is due to similar changes in the cell size and number regardless of early or late onset of overweight/obesity. In EOO, ESAT mass is larger due to a further increase in fat cell volume and number, but the resulting magnitude of slight hypertrophic morphology is almost the same in both overweight/obesity conditions.

**Fig. 3:**
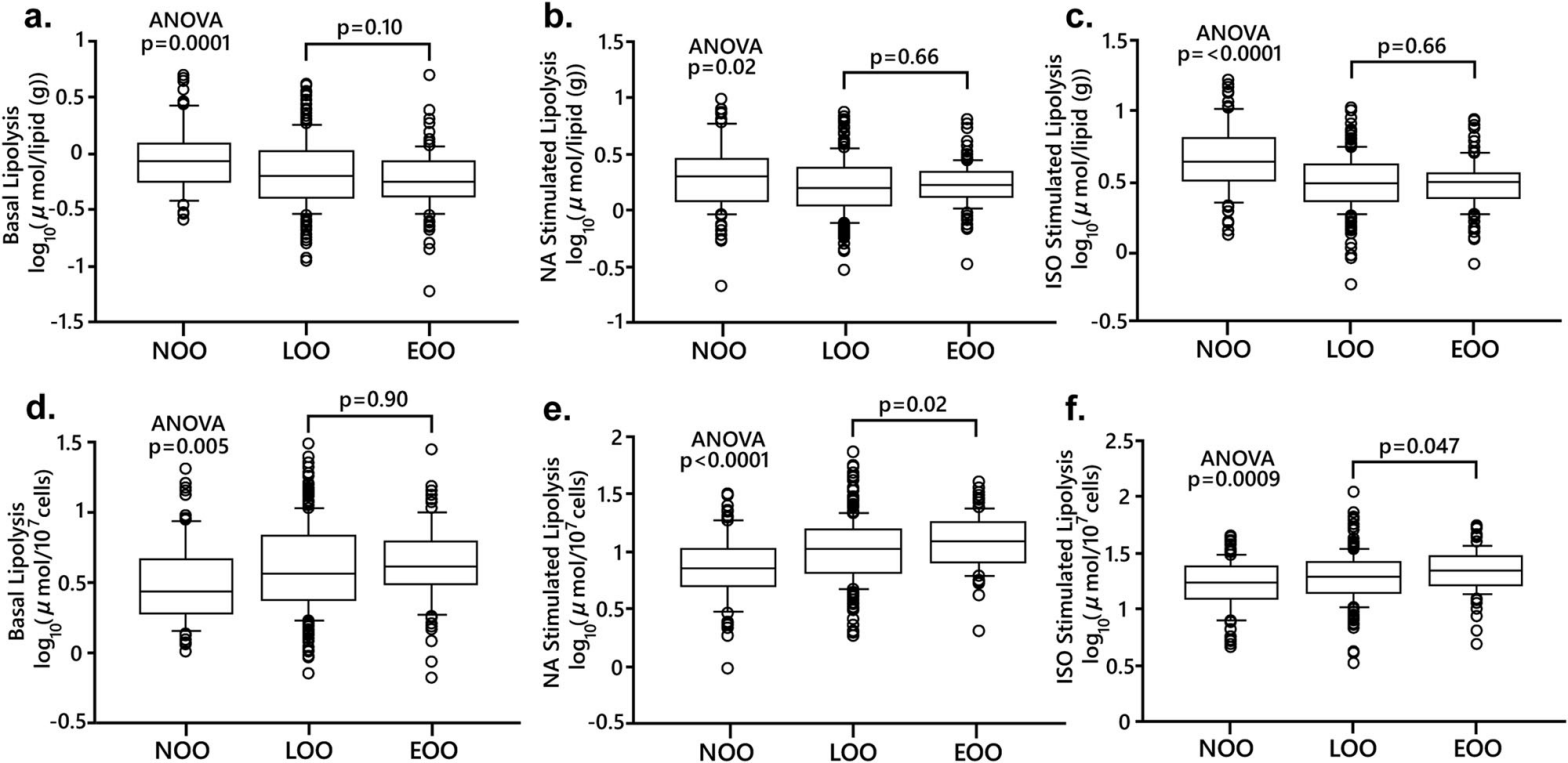
Findings with lipolysis (glycerol release) in abdominal subcutaneous fat cells. **a**–**c** Is lipolysis expressed per lipid weight. **d**–**f** Is lipolysis expressed per number of fat cells. Lipolysis is (10) log µmoles of glycerol/2 h. Values are expressed as box plots. See legend to Fig. 2 for further details.

Lipolysis data are displayed in Fig. 3. It is well known that comparisons between individuals with or without obesity is dependent on how lipolysis is expressed. Thus, when expressed per lipid weight, basal and stimulated lipolysis were lower in the overweight/obese state (Fig. 3a–c). There was, however, no differences between EOO and LOO. When lipolysis was expressed per number of fat cells the basal and stimulated rates were higher in overweight/obesity and for the catecholamines this was slightly more pronounced in EOO (Fig. 3d–f). To further explore the latter difference in lipolysis the overweight/obesity data were subjected to multiple regression using several factors which may independently influence lipolysis (age, sex, total body fat, waist-to-hip ratio, fat cell volume and onset of overweight/obesity) (Table S2). In this model only fat cell volume contributed to the differences in catecholamine stimulated lipolysis/number of fat cells.

To validate the clinical outcome findings at follow up, we constructed multiple regression models using: BMI, BMI increase per year, fat cell size and abdominal subcutaneous or visceral fat mass as dependent factors. As independent factors we put together current age, sex, initial BMI (at 18 years of age) and early/late onset of overweight/obesity. Age, sex, and initial BMI independently contributed (*p* < 0.0001–0.019) to the variations in current BMI, BMI increase/year and subcutaneous fat (*r*^2^ for the whole models was 0.40–0.54). Early/late onset of overweight/obesity had no important contribution in any of these models (*p* = 0.27–0.59). To further analyze the outcome for visceral fat and fat cell size we excluded early/late onset of overweight/obesity from the models and used all subjects (thus including lean people as well). Age, sex, and initial BMI contributed independently to the variations in visceral fat (*p* < 0.0001–0.0015; *n* = 409; *r*^2^ for the whole model = 0.11). Initial BMI (*p* < 0.0001) and sex (*p* = 0.003) contributed independently to the variations in fat cell size (*r*^2^ for the whole model = 0.10; *n* = 436).

We also examined highest and lowest self-reported BMI after 18 years of age. The difference between these measures were 5.3 ± 3.2, 12.3 ± 6.2, and 13.2 ± 5.5 kg/m^2^ for always lean, LOO and EOO, respectively (*p* < 0.000 by ANOVA). However, LOO and EOO did not differ from each other (*p* = 0.24 by *t*-test).

We finally made subgroup analyses. Excluding the few subjects on insulin or beta blockers did not impact importantly on the findings. Additionally, all data in the tables and Figs. 2 and 3 were analyzed in men and women separately. The exception was dicotomal variables in Table 1 because of a small number of subjects with indicated disease. Almost all analyses gave similar outcomes in either sex apart from the following: waist-to-hip ratio and visceral fat were slightly higher in males with EOO than LOO (*p* = 0.002 and 0.02, respectively), the difference in fat cell volume between LOO and EOO in women only reached *p* = 0.09, the differences in lipolysis between LOO and EOO were only apparent in men (*p* = 0.03 for noradrenaline and *p* = 0.055 for isoprenaline).

## Discussion

This large study sheds new light on the impact of early or late onset of overweight/obesity on human WAT. Although BMI and total body fat levels were higher in those with the early onset form, despite being younger than those with late onset there was no differences between the two conditions regarding putative cellular mechanisms behind adipose expansion (lipolysis, fat cell size/number or WAT morphology). One possible reason behind the differences could be the observation that there is a steeper increase in body weight in adulthood when excess body fat develops earlier than later in life. The differences in BMI persisted after correction for observation time. Our BMI results are in accordance with previous studies with early/late onset of excess body fat [12, 13].

When analyzing the clinical impact of BMI development, the subdivision into early or late onset of overweight/obesity had no significant impact on the outcome by itself. However, BMI at 18 years of age contributed significantly and independently to the variations in BMI, BMI increase per year, fat cell size and subcutaneous or visceral fat mass at follow up.

Several factors influence BMI. Our data suggest that expansion of subcutaneous WAT is the major cause of the difference between EOO and LOO because visceral fat was similar between these two groups in women and only slightly different in men. A minor difference in lean body mass also contributed to the BMI difference between EOO and LOO. The increased amount of body fat in overweight/obesity, at least in the abdominal subcutaneous WAT is explained by an increase in both fat cell size and number. In absolute terms these values were higher in EOO than LOO, which is likely due to the larger ESAT mass in the former group. Interestingly, the increase in BMI over time from 18 years of age positively correlated with fat cell size/number in all groups. When WAT morphology was determined quantitatively (delta value) only minor differences between the three groups were observed. The two forms of overweight/obesity were characterized by a very similar slight degree of hypertrophy. In those who were always lean, morphology tended to be hyperplastic. This is in accordance with previous results demonstrating a similar distribution of hypertrophy/hyperplasia over the whole range of BMI/total body fat [19, 20]. Therefore we suggest, firstly, that the same mechanisms are operating for expanding fat mass in early and later stages of life and, secondly, that fat mass in adulthood indeed can expand because of increases in fat cell number alongside fat cell size regardless of BMI status when leaving the childhood/adolescent period.

Impaired lipolysis may be a causal factor for development of excess body fat [9]. As demonstrated previously [21] the outcome of fat cell lipolysis studies in conditions of excess body fat is dependent on how the rate is expressed (per weight of lipids or number of fat cells). In accordance with these earlier studies lipolysis rates were decreased in overweight/obesity when expressed per lipid weight but increased when related to fat cell number. There was no influence of age on onset of overweight/obesity except for lipolysis data per fat cell number. However, this difference disappeared when results were corrected for the influence on lipolysis by fat cell volume. Furthermore, when subdivided according to sex, the minor differences in lipolysis between the obesity groups were only found in men. Thus, once elevated BMI is established, the duration of this condition does not further impact on disturbed lipolysis in an important way, at least not as regards to basal or catecholamine stimulated rates, which were determined in this study.

As discussed in detail [10, 11] it is unclear if body weight fluctuations such as weight cycling predispose for future overweight/obesity. Herein we obtained self-reported information about the highest/lowest BMI during adulthood. No difference between EOO and LOO was observed. Although this finding suggests that weight fluctuation is not a major cause of higher BMI in EOO compared to LOO, we cannot exclude that unrecognized differences in weight cycling following repeated dieting could play a role.

We also observed some differences between overweight/obesity groups in terms of fasting insulin and HDL-cholesterol parameters. However, these differences became non-significant in multiple regression analyses correcting for age, sex, and BMI. In addition, there was a small but significant difference in occurrence of diabetes between EOO and LOO. This may be related to the older age of the LOO group rather than the time of obesity onset. It should be noted that our study was not designed to examine the clinical impact of early or late onset of excess body fat. Other BMI related factors such as heredity/genetics, life style, pancreatic beta-cell function, incretins and microbiota not examined presently could be influenced by onset of overweight/obesity and impact on the clinical phenotype [22]. It is not likely that menopausal issues have influenced the results. In a recent longitudinal study we found that menstrual dynamics did not influence fat cell lipolysis [17]. It is somewhat surprising that the clinical profile only showed minor differences between the two obesity groups even though EOO had a longer duration of elevated BMI and had a more rapid increase in BMI over time in adulthood than LOO. Nevertheless, it might be of clinical value to encourage young people with high BMI to avoid a further BMI increase when getting older.

Based on present and previous results we propose the following model for development of excess body fat. Irrespective of an early or late start, fat mass expands by a combination of increased number and size of the fat cells. This results in more subcutaneous WAT when overweight/obesity develops earlier than later in life. Visceral fat mass expansion appears to be less influenced by the time of onset of elevated BMI. In addition, the putative mechanisms behind increases in fat mass in terms of adipose cellularity and fat lipolysis are not influenced in an important way by time of onset of fat mass expansion. The same is true for WAT morphology. We are aware our speculation is based on a cross-sectional examination and retrospective information about BMI at a single early time point. Preferably the idea should be validated by repeated laboratory examinations starting in adulthood and continued until very late stages in life.

This study has some limitations. We did not directly examine visceral fat cells and only subcutaneous fat cells from the abdominal region were investigated. It is possible that lower (gluteal/femoral) subcutaneous WAT is developed differently than upper (abdominal) subcutaneous fat. Differences in lipolysis and fat cell size between these two regions have been reported but they are only of a quantitative nature [23]. We have no information about adipose tissue or clinical variables besides BMI prior to the current laboratory investigation. Therefore, the results may above all relate to the present adult status of obesity/overweight. BMI at 18 years of age and BMI fluctuations were based on self-report. It is not possible for practical reasons to subject people to frequent laboratory measures of BMI from childhood to late stages in life. However, the self- reported body weight seems to be valid according to the quality check, at least for BMI at 18 years of age and using weighing at home. Other lipolysis regulating hormones besides catecholamines were not investigated. On the other hand, unlike for catecholamines there is no available information from prospective studies suggesting a causal role of such hormonal regulation in the expansion of fat mass. Finally, LOO and EOO were not perfectly matched for some parameters that may have influenced the findings with adipose tissue. However, we believe that the use of multiple regression to correct for such differences to some extent strengthens the validity of our results. It is in theory possible to also use propensity score matching to closer balance the groups. Although, this would decrease the number of comparable EOO and LOO subjects considerably and lower the statistical power. Using multiple regression we can investigate all subjects, allowing for a higher-powered study.

What are the mechanisms behind different amounts of subcutaneous WAT between LOO and EOO? As mentioned, they are probably not due to adipogenesis which determines fat cell size/number. Genetic/epigenetic factors or socioeconomic/behavioral factors not examined herein could play a role. Hormones could also be involved. Interestingly in a small subgroup we found that circulating leptin levels were almost 40% higher in EOO than LOO. This might impact on lipolysis, lean body mass and subcutaneous WAT as demonstrated previously [24–26].

In conclusion, subjects with early or late onset of overweight/obesity have similar disturbances in WAT function as regards cellularity, morphology, and lipolysis except for some minor differences in visceral fat mass and lipolysis in men. The most important difference is more excessive subcutaneous WAT accumulation in the early onset form which may at least in part be due to a more rapid accumulation of body fat in juvenile obesity.

## Supplementary information

Supplementary Tables

## Data Availability

All data that support the findings are available on request to the corresponding authors within reason. Material and correspondence requests should be made to the corresponding author.
