# Supplementary material for: Subcutaneous adipose tissue expansion mechanisms are similar in early and late onset overweight/obesity
Source: Int J Obes (Lond). 2022 Feb 28;46(6):1196–203. doi: 10.1038/s41366-022-01102-6 (PMC9151387; doi:10.1038/s41366-022-01102-6)
Supplement: Supplementary file 1 — Supplementary Tables [file 41366_2022_1102_MOESM1_ESM.docx]

| ***Factor*** | ***Fasting plasma HDL-cholesterol*** | | | | ***Fasting serum insulin*** | | | |
| --- | --- | --- | --- | --- | --- | --- | --- | --- |
|  | *Beta- coefficient* | *Standard error* | *95%, C.I.* | *p-value* | *Beta-coefficient* | *Standard error* | *95%, C.I.* | *p-value* |
| Age | 0.005 | 0.002 | 0.002 – 0.008 | 0.0008 | 0.002 | 0.04 | 0.74 – 0.078 | 0.96 |
| Sex | -0.24 | 0.038 | -0.308 – -0.160 | <0.0001 | 4.3 | 0.93 | 2.5 – 6.2 | <0.0001 |
| Body mass index | -0.011 | 0.003 | -0.009 – 0.005 | 0.0001 | 10.9 | 0.072 | 0.6 – 0.9 | <0.0001 |
| Early/late onset obesity | 0.013 | 0.041 | -0.066 – 0.092 | 0.74 | 1.1 | 0.98 | -0.8 – 3.0 | 0.27 |

**Table S1.** Role of different factors for variations in fasting HDL-cholesterol and insulin in obesity. A multiple regression model including age, sex, body mass index and onset of obesity was used. For the whole regression model r^2^ was 0.15 and 0.28 for HDL-cholesterol and insulin, respectively (p<0.0001).

| ***Factor*** |  | | ***Noradrenaline lipolysis per cell*** | | |  | ***Isoprenaline lipolysis per cell*** | | | |
| --- | --- | --- | --- | --- | --- | --- | --- | --- | --- | --- |
|  | *Beta-coefficient* | *Standard error* | | *95% C.I.* | *p-value* | *Beta-coefficient* | | *Standard error* | *95% C.I.* | *p-value* |
| Percent body fat | 0.005 | 0.10 | | -0.2 – 0.2 | 0.96 | 0.1 | | 0.13 | -0.2 – 0.4 | 0.45 |
| Fat cell volume | 1.9 | 2.8 | | 13 – 24 | <0.0001 | 25 | | 3.6 | 18 – 32 | <0.0001 |
| Early/late onset overweight/ obesity | -0.4 | 1.2 | | -2,7 – 1.9 | 0.75 | -0.07 | | 1.5 | -3.7 – 2.2 | 0.63 |
| Waist-to-hip ratio | 3.9 | 7.3 | | -10.7 – 18.4 | 0.60 | -4.6 | | 9.5 | -23.4 – 14.2 | 0.63 |
| Sex | 1.6 | 1.8 | | -1.9 – 5.2 | 0.37 | 3.3 | | 2.3 | -1.2 – 7.9 | 0.15 |
| Age | 0.05 | 0.05 | | -0.04 – 0.14 | 0.32 | 0.09 | | 0.06 | -0.03 – 0.20 | 0.14 |

**Table S2.** Role of fat cell volume and co-factors for variations in lipolysis in different forms of overweight/obesity. A regression model including several factors which could influence lipolysis was used. For the whole model r^2^ was 0.21 for noradrenaline or isoprenaline (p<0.0001).
